# Intravitreal ranibizumab and dexamethasone implant injections as primary treatment of diabetic macular edema: simultaneously double protocol

**DOI:** 10.1038/s41433-020-0949-2

**Published:** 2020-05-12

**Authors:** Mahmut Kaya, Nilufer Kocak, Taylan Ozturk, Volkan Bolluk, Ziya Ayhan, Suleyman Kaynak

**Affiliations:** Department of Ophthalmology, Dokuz Eylul University School of Medicine, Izmir, Turkey

**Keywords:** Retinal diseases, Prognosis

## Abstract

**Purpose:**

To assess the 12-month efficacy and safety of simultaneously administered intravitreal dexamethasone implant (DEX implant) and ranibizumab (simultaneous double protocol) injections in comparison with ranibizumab monotherapy as the first-line treatment of diabetic macular oedema (DMO).

**Methods:**

Prospective, consecutive, clinical interventional study. Patients were randomized into two groups: 24 naive DMO patients (34 eyes) who received simultaneous double-protocol therapy and 22 DMO patients (34 eyes) who received ranibizumab monotherapy were included. Monthly ranibizumab (0.5 mg) was administered for the first 6 months and later on, an as-needed treatment basis. DEX implant injection was performed at any time during the loading dose of the three consecutive monthly injections of ranibizumab, and with as-needed reinjections of ranibizumab from 6th month onwards. Change in visual acuity was the primary efficacy endpoint. Secondary efficacy endpoints were a gain of ≥15 letters and a change in the central foveal thickness.

**Results:**

Mean BCVA increased from baseline to month 12 in the simultaneously double-protocol therapy group compared with the ranibizumab monotherapy group (21.6 versus 9.6 letters [*P* < 0.001]). The corresponding proportions of eyes gaining ≥15 letters were 60% versus 29.4% (*P* < 0.0001). Moreover, the mean reductions in the central foveal thickness were 413 versus 282 µm (*P* = 0.001). At 12 month, the simultaneous double-protocol therapy decreased a significant number of foveal cysts and subfoveal neuroretinal detachment compared with those by ranibizumab monotherapy.

**Conclusions:**

The simultaneous addition of DEX implant at any time during the three monthly loading doses of ranibizumab in patients with DMO significantly improved the visual outcomes and revealed superior anatomic outcomes than those with the ranibizumab monotherapy.

## Introduction

Diabetic macular oedema (DMO) is increasingly one of the major causes of vision loss in the working-age population; its prevalence rises with advancing stages of retinopathy and before the development of retinal ischemia [1, 2]. Although the pathophysiology of DMO is not fully known, vascular endothelial growth factor (VEGF) is thought to play an important role in the pathogenesis of the disease [3, 4]. Besides VEGF, there is evidence for other cytokines contributing to the pathogenesis. Further, it was shown that multiple chemokines and proinflammatory cytokines, such as interleukin (IL)-6, IL-8, matrix metalloproteinase, IL-1beta, angiopoietin-2, tumor necrosis factor-alfa, and intercellular adhesion molecule-1, play a major role in the pathogenesis of DME [5–7]. Effective therapies are needed to address these complex processes involved in the pathogenesis of DMO. The multimechanism pathophysiology of DMO suggests a role for combination therapies that could have the potential to target more than one signaling cascade.

Nowadays, anti-VEGF agents are suggested as the first-line choice for center-involved DMO [8]. Anti-VEGF agents for DMO are typically administered monthly until the macula is dry, and either as-needed or treat-and-extend treatment can be used thereafter. However, persistent DME was found in 32–66% of eyes after at least 6 months of monthly anti-VEGF therapy, according to Diabetic Retinopathy Clinical Research (DRCR) protocol T [9]. Therefore, alternative or adjunctive treatment strategies such as intravitreal steroid and anti-VEGF injections have been used for DME recently. Intravitreal steroid stabilizes the blood–retinal barrier, inhibits some cytokines involved in the pathogenesis, and plays a role in preserving retinal homeostasis by regulating the activity of Müller cells [10–12]. Furthermore, the most important advantage of intravitreal steroids is that it controls the inflammatory component of DMO, which is prevalent in the most severe and chronic forms of the disease. This helps to achieve a more dramatic reduction in DMO in its initial stages. Theoretically, the simultaneous use of intravitreal anti-VEGF agents and dexamethasone implant (DEX implant) may reduce macular edema more effectively and more quickly, and prevent the loss of visual acuity caused by DMO.

Recently, clinical evaluation of noninvasive specific retinal biomarkers of retinal inflammation in diabetic retinopathy (DR) and DMO has become an important topic. These include the presence of intraretinal hyperreflective foci (HRF), subfoveal neuroretinal detachment (SND), hard exudates, and large cystoid spaces on spectral domain optical coherence tomography (SD-OCT).

To the best of our knowledge, this is the first study on simultaneous protocol with intravitreal ranibizumab and DEX implant as primary therapy for DMO. In this prospective and randomized study, we investigated the efficacy and safety of simultaneous double protocol of intravitreal ranibizumab and DEX implant in comparison with ranibizumab monotherapy as the first-line treatment of DMO.

## Materials and methods

The Dokuz Eylul University’s ethics committee approved our study design and protocol. The patients were fully informed about the study and their informed consents were obtained. This prospective, randomized, and open-label study in naive patients with DMO was conducted in the retina clinics of the Department of Ophthalmology, Dokuz Eylul University, School of Medicine, from September 12, 2016, until May 25, 2018, for a minimum follow-up period of 12 months and was performed according to the tenets of the Declaration of Helsinki.

A participant could have one or two eyes included in the study. Participants with one study eye were randomly assigned with equal probability to one of the both groups, to receive either a combination of intravitreal ranibizumab (Lucentis®; 0.5 mg, Novartis Pharma AG, Switzerland), and DEX implant (Ozurdex; 0.7 mg, Allergan, Inc., Irvine, CA, USA), injection (simultaneous double protocol group) or intravitreal ranibizumab injections (ranibizumab monotherapy group). Participants with two study eyes had one eye randomly assigned to each group. Participants with two study eyes had one eye assigned randomly to simultaneously double protocol group and the other to ranibizumab monotherapy group. The study design is presented in Supplementary Fig. 1.

Naive male and female patients, at least 18 years old with type 2 diabetes mellitus, were included. Also included were those with decreased best corrected visual acuity [BCVA] of 20/40 or worse, Snellen equivalent using Early Treatment for Diabetic Retinopathy Study [ETDRS] charts) from DMO who had been treated either with intravitreal ranibizumab according to the user guide (a monthly injection for the first six consecutive doses, and then treated on as-needed/pro-re-nata [PRN] basis) or with simultaneous intravitreal DEX implant and the above intravitreal ranibizumab injection protocol (simultaneous double protocol) and the presence of DMO with ≥300 µm foveal intraretinal cystoid spaces (within 1000 µm of the center of the fovea), SND, intraretinal HRF (within 500 µm of the center of the fovea) or foveal exudates on SD-OCT (Supplementary Fig. 1). Exclusion criteria included a history of vitreoretinal surgery and/or including scleral buckling in the study eye; Grades III and IV or those with posterior subcapsular cataracts, eyes with thicker epiretinal membranes (ERM) who had central foveal involvement and vitreomacular traction; inflammation; active or suspected ocular or periocular infections; laser photocoagulation (panretinal or macular) in the study eye within 3 months; advanced glaucoma (baseline intraocular pressure [IOP] higher than 21 mmHg or use of more than one type of glaucoma medication); a history of steroid-induced IOP increase; previous treatment with antiangiogenic drugs (e.g., aflibercept, bevacizumab, and ranibizumab) in either eye. Patients with type 1 diabetes mellitus, uncontrolled hypertension, cerebrovascular accident or myocardial infarction within 3 months, and uncontrolled diabetes (glycosylated hemoglobin [HbA1c] > 12%) were also excluded. Patients who were pregnant or breastfeeding and with retinal or choroidal disease other than DR that could affect central foveal thickness (CFT) were also excluded.

A detailed ophthalmologic examination, including BCVA using ETDRS charts, slit-lamp biomicroscopy, tonometry, indirect fundus ophthalmoscopy, fluorescein angiography, and SD-OCT (Spectralis, Heidelberg Engineering, Heidelberg, Germany) were performed in all patients. CFT that was measured in the central 1 mm diameter circle of the ETDRS thickness map by Spectralis software was recorded. Participants in both treatment groups had assessment visits every 4 weeks for 12 months.

### Ranibizumab

In both groups, patients received monthly intravitreal ranibizumab injection during the first 6 months. Between months 6 and 12, all patients were allowed to receive “as-needed/pro-re-nata” (PRN) intravitreal ranibizumab. At each follow-up visit, investigators evaluated the study eye for re-treatment based on visual acuity and SD-OCT findings. A decrease in BCVA > 5 letters and/or a CFT > 300 μm were indications for re-treatment. In the maintenance of both BCVA improvement and decrease in CFT after the first 6 months, treatment was continued.

### DEX implants

In the simultaneous double protocol group, the first DEX implant injection was received along with three loading doses of ranibizumab injections. The patients were evaluated for re-treatment after every 6 months after the first DEX implant injection. At the 6th month, patients were allowed to receive the second dose of DEX implant in the presence of DMO with ≥300 µm foveal intraretinal cystoid spaces (within 1000 µm of the center of the fovea), SND, intraretinal HRF (within 500 µm of the center of the fovea) or foveal exudates on SD-OCT. Re-treatment with DEX implant was on a PRN basis. In 22 patients, the simultaneously double protocol group, the first DEX implant was performed simultaneously (at the same time points in all patients) with the first dose of intravitreal ranibizumab injection. Only in two patients, was the first DEX implant performed simultaneously with the second dose of intravitreal ranibizumab injection due to their health insurance problems.

All injections were administered in the operating room. A complete ophthalmologic examination and SD-OCT scans for the first year were performed monthly in all patients regardless of their treatment.

### Outcome measures

The primary efficacy endpoint was the change from baseline in BCVA in ETDRS letters at month 12. The secondary efficacy endpoints were (1) proportion of eyes that gained ≥15 letters from baseline, (2) change from baseline in CFT, as determined by SD-OCT.

### Statistical methods

All statistical analyses were performed using SPSS version 22.0 software (SPSS Inc, Chicago, IL). The descriptive data of the patients were expressed as the mean, standard deviation, or the percentages of variables. When the normality of distribution was checked by Kolmogorov–Smirnov analysis, a normal distribution was detected, and the significance was determined by using parametric tests. To test for differences between the groups and to adjust for age and gender, an analysis of covariance was performed with diagnosis and sex as fixed variables and age as a covariate. Power analysis was performed using the Power Analysis and Sample Size 2008 Statistical Software (NCSS, Kaysville, UT). A sample size of 58 eyes was calculated to ensure that the minimum required size was within a 95% confidence interval (CI) and 5% of the true proportion. A *P* value of <0.05 was considered statistically significant.

## Results

A total of 68 eyes of 46 patients were assessed for eligibility. This was a prospective randomized study including all patients undergoing simultaneous double protocol (34 eyes of 24 participants) or ranibizumab monotherapy protocol (34 eyes of 22 participants). An age and gender-matched group who had received ranibizumab monotherapy injections 9–12 times over a 12-month period was selected as the control group. The mean age of the participants was 65 ± 10 years (range, 49–86 years); 52% were men. All our participants had type 2 diabetes with a mean disease duration of 14 ± 6 years. At baseline, the mean visual acuity letter score was 50.7 ± 17.9 (Snellen equivalent, ~20/100), and the mean central subfield thickness was 597 ± 250 μm. Patients within the two study arms were matched for age, gender, baseline visual acuity, baseline central subfield thickness, HbA1c levels, and systemic hypertension (*P* > 0.05). Baseline demographic and clinical characteristics for participants in the two treatment groups are presented in Table 1, which were similar.

**Table 1:** Patient demographic and baseline characteristics.

	Treatment groups		
Characteristic	Simultaneous double protocol	Ranibizumab monotherapy protocol	*P*
Patients, *n*			
Mean age (years) ±SD	64.6 ± 10.5	66.2 ± 8.8	0.457
Male sex, *n* (%)	12 (50)	12 (54.5)	0.760
Hypertension, *n* (%)	8 (33.3)	10 (45.5)	0.716
Type of diabetes, *n* (%)			
Type II	24 (100)	22 (100)	0.627
Mean diabetes duration (years) ±SD	13.6 ± 5.1	14.2 ± 5.8	0.154
Mean duration of insulin use (years) ±SD	8.3 ± 3.3	8.7 ± 3.8	0.258
Mean HbA1c % ± SD	7.0 ± 0.8	7.0 ± 0.6	0.677
Eyes, *n*			
Lens status			
Pseudophakic, *n* (%)	12 (33.3)	10 (29.4)	0.268
Mean ETDRS letters score ± SD	48 ± 23	52 ± 14	0.416
Mean approximate Snellen equivalent			
≤20/200, *n* (%)	14 (41.2)	10 (29.4)	0.532
>20/200 but <20/40, *n* (%)	18 (52.9)	22 (64.7)	0.503
≥20/40, *n* (%)	2 (5.9)	2 (5.9)	0.899
Central foveal thickness			
≤350 µm	4 (11.8)	8 (23.5)	0.305
>350 µm but <500 µm	13 (38.2)	10 (29.4)	0.740
≥500 µm	17 (50)	16 (47.1)	0.623

### Visual acuity outcomes

**Fig. 1:**
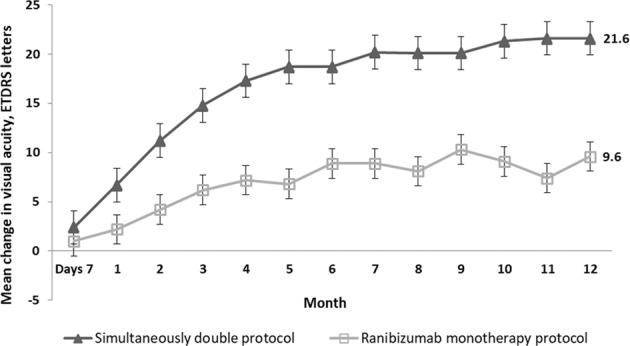
Visual outcomes from baseline to month 12. Mean ± standard deviation (SD) change in Early Treatment Diabetic Retinopathy Study (ETDRS) letter chart scores from baseline through month 12.

Figure 1 highlights the mean gained ETDRS letters monthly in the two treatment groups over the first year of the study. BCVA letter score improved at each follow-up resulting in significantly higher rates compared with baseline by 1 month for both the simultaneous double protocol group (+6.5 letters; 95% CI: 2.5–10.5) and the ranibizumab monotherapy group (+2.2 letters; 95% CI: 0.4–4.1) (*P* = 0.002 and *P* = 0.018, respectively), and continued to improve steadily up to the last follow-up visit (*P* < 0.05, all time points).

**Fig. 2:**
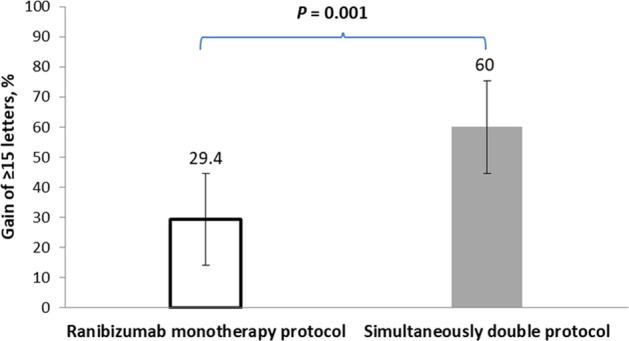
Visual acuity letter score after 12-month. Proportion of eyes that gained ≥15 letters from baseline to month 12.

Compared with the baseline, the mean improvement in BCVA was +20.5 letters at month 6 and +21.6 letters at month 12 for the simultaneous double protocol group, and +8.9 letters at month 6 and +9.6 letters at month 12 for the ranibizumab monotherapy group (*P* = 0.002 at 6 months, *P* < 0.001 at 12 months; Fig. 1). In the simultaneous double protocol group, 56.7% of patients gained at least 15 letters from baseline at month 6 compared with 26.5% of patients in the ranibizumab monotherapy group (*P* = 0.014). In the simultaneous double protocol group, 60% of patients gained at least 15 letters from baseline at month 12 compared with 29.4% of patients in the ranibizumab monotherapy group (*P* = 0.001; Fig. 2).

More than half of the patients in the simultaneously double protocol group reached a Snellen BCVA of ≥20/40 at month 12 compared with the ranibizumab monotherapy group (*P* = 0.013; Supplementary Fig. 2**:** full image is provided at the end of Supplementary file). A statistically significant improvement in vision was observed with the simultaneous double-protocol therapy when compared with the ranibizumab monotherapy as early as 8 months after injections (*P* < 0.001).

### Anatomic outcomes

**Fig. 3:**
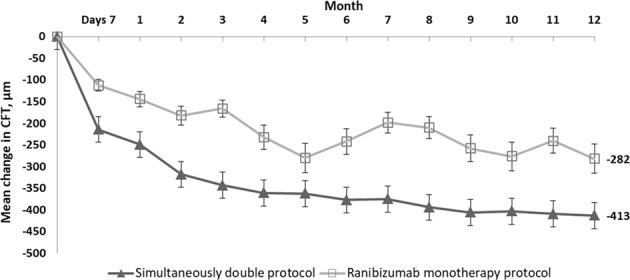
Mean Change in Central Foveal Thickness (CFT) over time. Mean change from baseline in CFT at each study visit through month 12.

Figure 3 demonstrates mean CFT changes at different follow-ups in relation to the baseline in the two groups. Comparing with the baseline, the CFT reduction was statistically significant in both the simultaneous double protocol group and the ranibizumab monotherapy group at all follow-ups. Nonetheless, the reduction in CFT was more significant in the simultaneous double protocol group than that in the ranibizumab monotherapy group. Differences between the simultaneous double protocol and ranibizumab monotherapy groups were statistically significant at 2 months and at each point thereafter.

Table 2 shows the differences in the morphological findings between the study groups. Baseline means “number of foveal cysts at 1000 µm, ≥300 µm” and this value decreased significantly from 2.7 ± 1.3 to 0.9 ± 1.1 in the simultaneous double protocol group at month 12 (*P* < 0.0001). However, the mean “number of foveal cysts at 1000 µm, ≥300 µm” reduction at month 12 was not significant in the ranibizumab monotherapy group (*P* = 0.129). At month 12, 60% of patients in the simultaneous double protocol group had SND completely resolved (*P* < 0.0001). Conversely, in the ranibizumab monotherapy group, the percentage of patients with SND at month 12 was not significantly different when compared with that observed at the baseline (*P* = 0.097). At month 12 follow-up, the mean intraretinal HRF (at a distance of 500 μm) showed a statistically significant decrease in both the simultaneous double protocol and the ranibizumab monotherapy groups (*P* < 0.0001 and *P* = 0.001, respectively). In addition, there was no statistically significant increase in the percentage of patients with ERM in both groups at month 12 (*P* = 0.161 and *P* = 0.051 in the simultaneous double protocol and ranibizumab monotherapy groups, respectively).

**Table 2:** Differences in SD-OCT morphological findings between the study groups at baseline and 12 months.

	Simultaneous double protocol	Ranibizumab monotherapy protocol	*P*
SD-OCT findings at baseline			
Number of foveal cysts (at 1000 µm) ±SD, ≥300 µm	2.7 ± 1.3	2.2 ± 1.3	0.073
Subfoveal neuroretinal detachment, *n* (%)	18 (60)	18 (53)	0.577
Number of intraretinal hyperreflective foci ± SD (at 500 µm)	10.8 ± 3.5	10 ± 4.4	0.428
Epiretinal membrane, *n* (%)	6 (20)	5 (15)	0.582
SD-OCT findings at 12 months			
Number of foveal cysts (at 1000 µm), ≥300 µm	0.9 ± 1.1	1.6 ± 1.3	**0.034**
Subfoveal neuroretinal detachment, *n*	0	12 (35)	**<0.0001**
Number of intraretinal hyperreflective foci ± SD (at 500 µm)	6.1 ± 3.5	6.8 ± 3.5	0.097
Epiretinal membrane	10 (33)	12 (35)	0.872

Two-tailed *t* test, *P* < 0.05 was considered statistically significant.

Two-tailed *t* test, *P* < 0.0001 was considered extremely significant.

Multivariate linear regression analysis showed that improvement in vision was significantly associated with low number of foveal cysts (*r* = 0.214, *P* = 0.004) and reduction in SND (*r* = 0.308, *P* = 0.006) at final visit.

### Number of treatment

The mean numbers of injections for the simultaneous double protocol group were 8.3 of ranibizumab and 1.7 of DEX implant, and 10.4 of ranibizumab for the ranibizumab monotherapy group over the 12 months. Twenty-one eyes (61.8%) needed second DEX implant injection; thirteen eyes (38.2%) needed first injection.

### Ocular adverse events

Cataract and increased IOP were the ocular adverse events. No cases with treatment-related retinal detachment or endophthalmitis were detected in the present study.

#### Intraocular pressure

At least 5 mmHg of IOP elevation from baseline was seen in 35.3% (12/34) of eyes treated with DEX implant and in 18% (6/34) of those treated with ranibizumab during follow-up visits. Furthermore, during the whole study, an IOP of more than 25 mmHg at least once was demonstrated in eight eyes. Twelve eyes with DEX implants revealed high IOPs. Patients with increased IOPs were treated successfully with either observation or topical antiglaucoma medications.

#### Cataract progression

The percentage of pseudophakic eyes was 38.2% (26/68) at baseline: 12 eyes (35.3%) were treated with simultaneous DEX implant and ranibizumab, whereas 10 eyes (29.4%) were treated with ranibizumab alone. We could detect an increase in cataract density by two grades or more from the baseline in 11.8% (4/34) of eyes in the simultaneously double protocol group and in 5.9% (2/34) of eyes in the ranibizumab monotherapy group. During the study, four eyes in the simultaneous double protocol group and two eyes in the ranibizumab monotherapy group underwent cataract surgery in the study eye. Their postoperative results were included in the final analysis.

### Systemic adverse events

The DEX implant and ranibizumab injections were systemically well tolerated in both groups. Moreover, no systemic serious adverse event was observed at any follow-up visits.

## Discussion

The present clinical study revealed that treatment with intravitreal ranibizumab alone or with DEX implant injections has significant beneficial effects on the anatomical and functional outcomes of DMO. The simultaneous double-protocol therapy group was superior to ranibizumab monotherapy protocol group across all measures of vision improvement. Although there was a statistically significant increase in the primary efficacy endpoint (change from baseline in BCVA at 12 months) in the ranibizumab monotherapy protocol group, the increase in the simultaneous double-protocol therapy group was observed very rapid and better. The results demonstrate that simultaneous intravitreal DEX implant and ranibizumab injection is superior to ranibizumab monotherapy and results in both significant visual acuity gains and prevention of severe visual acuity loss. In addition to the primary efficacy outcome, the eyes treated with simultaneous double protocol had a 2 times greater chance of gaining 15 or more letters and 1.5 times greater chance of attaining a Snellen equivalent BCVA of ≥20/40 at month 12 than that by the eyes treated with ranibizumab monotherapy protocol. Although the CFT reduction observed in relation to the baseline measurement was significant at 4 weeks in both the simultaneous double protocol and the ranibizumab monotherapy protocol groups, the differences among the groups were statistically significant at 2 months and thereafter.

The analyses of data collected in the RISE/RIDE (ranibizumab) [13] and VIVID/VISTA (aflibercept) [14] clinical trials in the treatment of DMO had reported clear superiority to control groups in terms of the outcomes in visual and anatomical parameters. Moreover, the European Society of Retina Specialists [8] recommended anti-VEGF therapy as the first-line therapy for the treatment of DMO. However, a negative effect on visual and anatomical outcomes may be observed in some patients as DMO can chronically persist despite anti-VEGF therapy [9, 15]. Addition of DEX implant to anti-VEGF therapy with ranibizumab might solve the problem of the residual DMO [15].

Based on the data that exist thus far, the DEX implant has been used as a second-line treatment option for the management of DMO [8]. Steroid therapy is effective as it has anti-inflammatory, antipermeability, and angiostatic effects for the successful treatment of DMO. Further, intravitreal steroid therapy has also been a popular treatment as it quells the activity of multiple inflammatory mediators [16]. One of the most challenging topics in the management of DMO is whether the addition of DEX implant in patients treated with ranibizumab provides any additional benefit. In particular, the absence of the ellipsoid layer, the presence of disorganization of the retinal inner layers (DRIL) [17], an increase in the number of HRS in the outer retinal layers around the intraretinal cystoid spaces [18], the presence of foveal exudates [19], the presence of SND [20], and the presence of large cystoid spaces in the fovea (≥300 µm) [21] on baseline SD-OCT are highly related to the final visual function in DMO; therefore, these findings can be considered as the imaging biomarkers of retinal inflammatory response. Patients who have any of these inflammatory biomarkers on SD-OCT and/or poor visual acuity can achieve improved functional and anatomical results using simultaneous DEX implant and ranibizumab therapy compared with those using ranibizumab monotherapy. In the present study, we preferred to inject a single implant of dexamethasone at any time during the loading dose of three consecutive monthly injections of ranibizumab followed by monthly intravitreal ranibizumab injections until the 6th month, and thereafter as-needed (PRN) protocol was used. The 12 months results of this study demonstrated that the simultaneous double-protocol therapy significantly improved visual outcomes and significantly decreased CFT compared with ranibizumab monotherapy. Moreover, the morphological changes usually associated with active inflammation such as, SND, HRF, foveal hard exudates, or large foveal cystoid spaces, decreased more rapidly from the foveal center after the 2 initial months of the double-protocol therapy and remained stable over 12 months. We identified the presence of SND, large cystoid spaces in the fovea as biomarkers that predict better visual outcome after the simultaneous double-protocol therapy in eyes with DMO.

The simultaneous double-protocol therapy group was found to have a less macular thickness and fewer fluctuations in vision than those in the ranibizumab monotherapy group in our study. We speculated that the severity of fluctuations had negative effects such as ganglion cell loss, and DRIL formation on retinal morphology. Future studies should be performed to confirm the association between anti-VEGF therapy and these fluctuations in a larger number of patients.

There are few studies on combined DEX implant and anti-VEGF treatment for DMO [22, 23]. Protocol U of the DRCR Network study was phase 2 randomized clinical trial, multicenter investigation of 129 eyes with persistent DMO. Eyes received at least three anti-VEGF injections before a run-in phase, which consisted of additional monthly ranibizumab 0.3 mg injections during a period of 12 weeks. Eyes with persistent DMO were then randomized either a combination of ranibizumab and intravitreal DEX 0.7 mg injection (combination group) or sham and ranibizumab 0.3 mg injections (ranibizumab group). The researchers found no significant difference between groups in mean BCVA at 24 weeks (mean improvement in BCVA of 2.7 letters in the combination group and 3 letters in the ranibizumab group, *P* = 0.73). However, the combination therapy group demonstrated significantly reduced retinal thickening (–110 µm in CFT compared with –62 µm in the ranibizumab group; *P* < 0.01) [22]. In summary, Protocol U results showed that the improvement in visual acuity was not more in patients on intravitreal steroid added to continued ranibizumab therapy than that in patients on ranibizumab therapy alone for persistent DMO at 24 weeks. Besides, they stated that the decrease in retinal thickness was observed mostly in patients with the combination therapy. The fact that reduced macular thickening was significantly improved in the combination group without improvement in BCVA suggests that the addition of the dexamethasone implant may have occurred after photoreceptor death. The time until transition of therapy was long (at least six anti-VEGF injections prior to randomization), and these eyes were not treatment naive. Busch et al. [23] reported better functional and anatomical 24-month outcomes in eyes nonresponsive to anti-VEGF during the loading phase switched early to DEX implant compared with those continued on anti-VEGF agents alone. Eyes that received anti-VEGF therapy alone did not significantly improve vision or reduce their CFT at 12 or 24 months compared with baseline. Eyes switched from anti-VEGF therapy to steroids in the second year also showed significant functional and anatomical benefits in their study. In the current study, both improved visual acuity and reduced macular thickness were shown in the simultaneous double protocol group. In contrast to the Protocol U study, eyes in the simultaneous double protocol group received additional DEX implants during the loading dose of ranibizumab. At start of treatment, intensive anti-VEGF treatment and DEX implant led to better functional and anatomical outcomes than less intense treatment. In another study, Hernández-Bel et al. [24] reported that sequential intravitreal treatment in DMO starting with dexamethasone and followed by aflibercept was a reasonable alternative to treatment with aflibercept alone for reducing the burden of treatment over 12 months. A difference noted in comparison with our study was that the improvement in vision and the decrease in macular thickness were not statistically significant. The data from our study revealed that the simultaneous double-protocol therapy provided a more rapid and dramatic improvement of DMO and significant improvements in vision and macular thickness compared with the ranibizumab monotherapy.

Cost-effectiveness of treatments and their impact on quality of life are of critical importance when considering an appropriate treatment for DMO. A cost-effectiveness analysis accompanying the 1-year data of Protocol T revealed that aflibercept was not cost-effective versus ranibizumab for all patients using a model with a 1-year time horizon, but was cost-effective in patients with baseline VA of 20/50 or worse using a 10-year time horizon [25]. Real-world healthcare utilization of DMO cohorts who were treated with anti-VEGF or the DEX implant and using data from the Korean NHI claim database study was showed that the average annual eye-related medical cost of the DEX implant group was significantly lower than that of the anti-VEGF group during the study period, which was mainly due to decreased utilization of eye care-related injections [26]. Although both treatment arms have similar costs in our study, simultaneous DEX implant and ranibizumab treatment are more effective for improving vision in patients with DMO during long-term follow-up (12 months), suggesting that the DEX implant might be clinically beneficial as well as economically attractive.

Our study had several limitations. Perhaps the most important limitation of this prospective, randomized study includes a relatively small sample size. Another limitation was that it included subjects who had poor visual acuity and increased macular thickness. The number of patients enrolled in the study may be not sufficient to detect systemic serious adverse events. Finally, aflibercept is demonstrated to have superior visual acuity outcomes than those with ranibizumab in eyes with baseline visual acuities of 20/50 to 20/320 [9]. Further prospective studies may be designed for investigating the effects of aflibercept and DEX implant on DMO.

## Conclusions

Intravitreal ranibizumab monotherapy significantly improved macular edema and vision in patients with DMO. Furthermore, the simultaneous treatment with intravitreal DEX implant at any time during the three monthly loading injections of ranibizumab significantly improved the visual outcomes and revealed superior anatomical outcomes than those with ranibizumab monotherapy in DMO patients with inflammatory biomarkers on SD-OCT and/or poor visual acuity.

## Summary

### What was known before

- Nowadays, anti-VEGF agents are suggested as the first-line choice for center-involved DMO. Anti-VEGF agents for DMO are typically administered monthly until the macula is dry, and either as-needed or treat-and-extend treatment can be used thereafter. However, persistent DMO was found in 32–66% of eyes after at least 6 months of monthly anti-VEGF therapy, according to the Diabetic Retinopathy Clinical Research protocol T.

### What this study adds

- The results of our study demonstrated that simultaneous intravitreal dexamethasone implant and ranibizumab injection was superior to ranibizumab monotherapy and resulted in both significant visual acuity gains and prevention of severe visual acuity loss.

## Supplementary information

Supplementary Figure 1

Supplementary Figure 2

Supplementary Data
