# Supplementary figures and images for: Intravitreal ranibizumab and dexamethasone implant injections as primary treatment of diabetic macular edema: simultaneously double protocol
Source: Eye (Lond). 2020 May 12;35(3):777–85. doi: 10.1038/s41433-020-0949-2 (PMC8027799; doi:10.1038/s41433-020-0949-2)

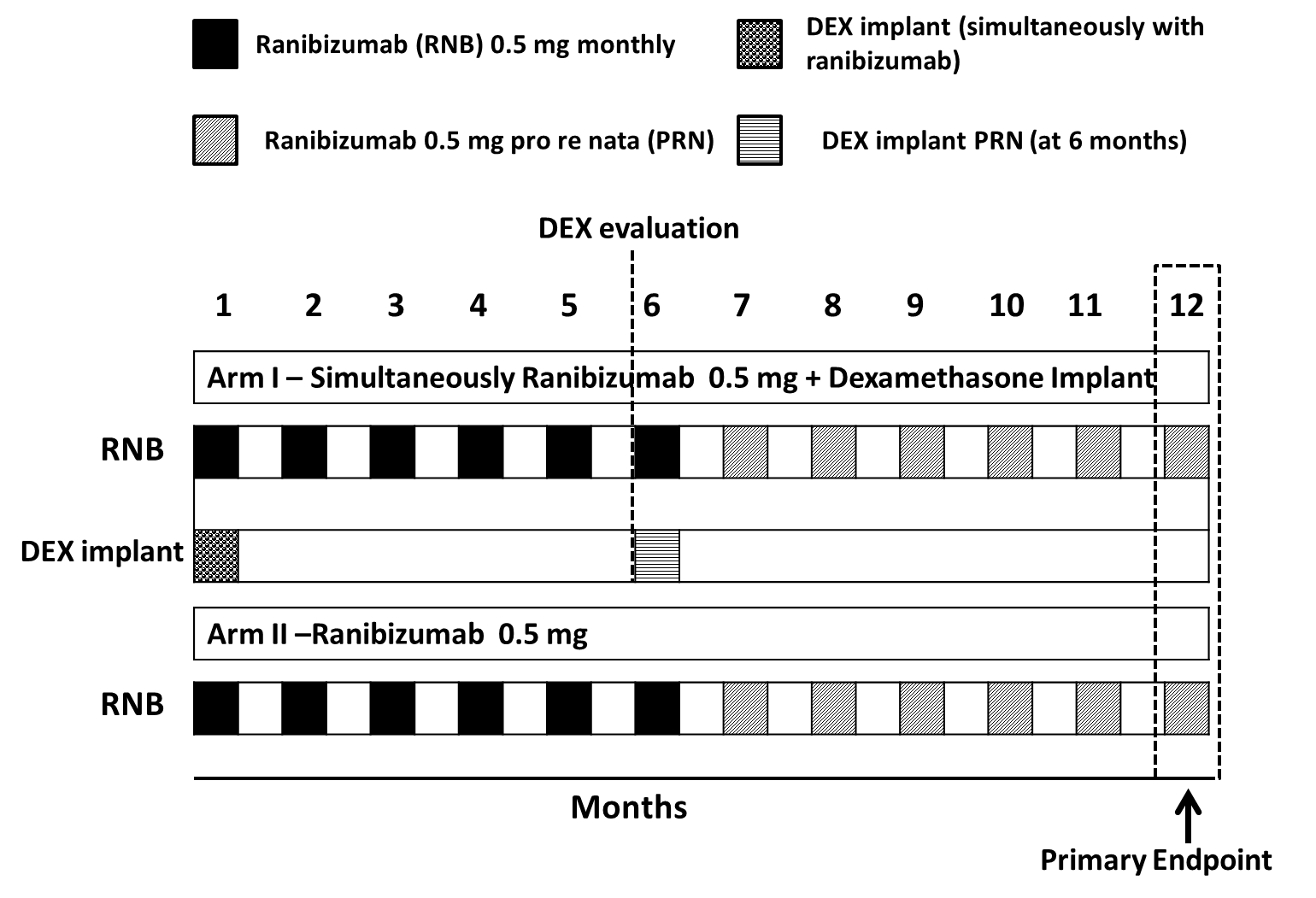

Supplement: Supplementary file 1 — Supplementary Figure 1 [file 41433_2020_949_MOESM1_ESM.tif]

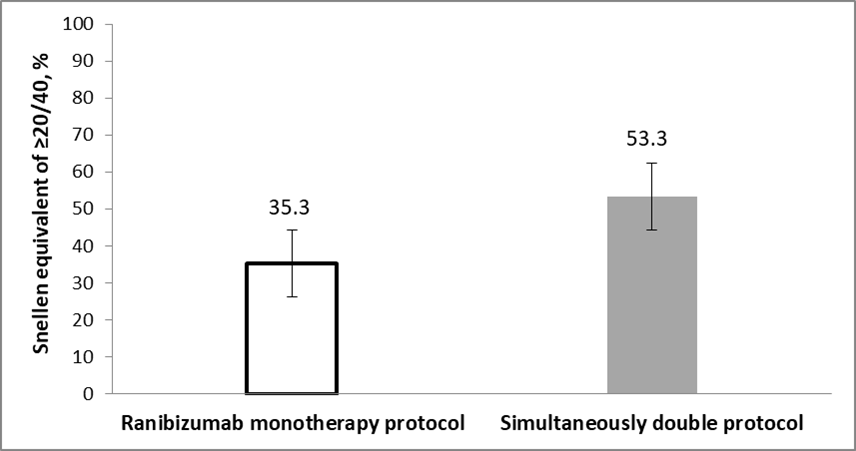

Supplement: Supplementary file 2 — Supplementary Figure 2 [file 41433_2020_949_MOESM2_ESM.tif]
