# Supplementary material for: Intravitreal ranibizumab and dexamethasone implant injections as primary treatment of diabetic macular edema: simultaneously double protocol
Source: Eye (Lond). 2020 May 12;35(3):777–85. doi: 10.1038/s41433-020-0949-2 (PMC8027799; doi:10.1038/s41433-020-0949-2)
Supplement: Supplementary file 3 — Supplementary Data [file 41433_2020_949_MOESM3_ESM.docx]

**Fig. S1 (Supplementary file).** Study design.

**Fig. S2 (Supplementary file).** Proportion of eyes that gained ≥20/40 letters from baseline to month 12.
